# Prevalence and Exposure Assessment of Aflatoxins Through Black Tea Consumption in the Multan City of Pakistan and the Impact of Tea Making Process on Aflatoxins

**DOI:** 10.3389/fmicb.2020.00446

**Published:** 2020-03-31

**Authors:** Amir Ismail, Saeed Akhtar, Muhammad Riaz, Yun Yun Gong, Michael N. Routledge, Iqra Naeem

**Affiliations:** ^1^Institute of Food Science and Nutrition, Bahauddin Zakariya University, Multan, Pakistan; ^2^School of Food Science and Nutrition, University of Leeds, Leeds, United Kingdom; ^3^School of Medicine, Faculty of Medicine and Health, University of Leeds, Leeds, United Kingdom; ^4^School of Food and Biological Engineering, Jiangsu University, Zhenjiang, China

**Keywords:** aflatoxins, tea, health, cancer, estimated daily intake, beverage

## Abstract

Aflatoxins are the highly toxic secondary metabolites of certain fungi, being mainly produced by *Aspergillus flavus* and *Aspergillus parasiticus*. Aflatoxins are classified as group 1 category carcinogens by the International Agency for Research on Cancer (IARC). A large number of food commodities are reported to be contaminated with aflatoxins. Tea is the world’s second most consumed beverage and the consumption of tea is increasing day by day. Besides being a source of several health promoting substances, tea leaves are also reported to be contaminated with aflatoxins. However, not a single study is reported from Pakistan regarding the level of aflatoxins in commercially available black tea samples. The current study aimed to quantify the level of aflatoxins in commercially available branded and non-branded black tea samples. The estimated daily intake (EDI) of aflatoxins through branded and non-branded black tea consumption and the health risk assessment based on margin of exposure (MOE) approach was assessed. Furthermore, the impact of local tea making processes on the concentration of aflatoxins in tea beverage (filtrate) was also investigated.

## Introduction

Tea, the world’s second most commonly consumed beverage after water is produced by the infusion of *Camellia sinensis* leaves that are native to Southeast Asia. China, India, Kenya, Sri Lanka, Vietnam, Turkey, and Indonesia are the leading tea producers (Heck and de Mejia, 2009). The world’s total tea production is estimated to rise from 2.9 million tons in 1994 to 6.1 million tons in the year 2017. The annual consumption of tea is estimated to be around 273 billion liters and is forecasted to rise to 297 billion liters by the year 2021 (FAOSTAT, 2017; Statista, 2019). Depending on the degree of fermentation, tea is generally categorized into three major categories, i.e., black, green, and oolong. Black tea is the most commonly consumed tea worldwide and estimated to be around 78% of total tea produced (Soni et al., 2015). Black tea is a rich dietary source of polyphenols such as theaflavins, catechins, and thearubigin, which have the potential to influence the pathogenesis of several chronic disorders owing to their antioxidant, anti-proliferative, anti-inflammatory, antiviral, antibacterial, anti-mutagenic, cardio-protective, and neuroprotective effects (Da Silva Pinto, 2013). Moreover, black tea is also reported to contain appreciable quantities of some essential micronutrients such as fluoride, copper, and manganese (Zhang et al., 2018b). However, despite being a source of several health-promoting components, tea samples are also reported to be contaminated by a number of toxicants, the most critical among which are the mycotoxins, especially aflatoxins (Viswanath et al., 2012; Pouretedal and Mazaheri, 2013).

Mycotoxins are the secondary metabolites of certain species of fungi that may infect the crops intended for human consumption or use as animal feed. More than 450 different types of mycotoxins are reported to date, but the most toxic among all mycotoxins are aflatoxins. The major producers of aflatoxins are of the genus *Aspergillus*, primarily *Aspergillus flavus* and *Aspergillus parasiticus*. More than 17 different types of aflatoxins are reported, the most toxic as well as the most reported in the food and feed items are aflatoxin B_1_ (AFB_1_), aflatoxin B_2_ (AFB_2_), aflatoxin G_1_ (AFG_1_), and aflatoxin G_2_ (AFG_2_). The sum of these four naturally existing aflatoxins is collectively known as total aflatoxins (TAF). Aflatoxins are reported to be hepatotoxic, teratogenic, mutagenic, neurotoxic, immuno-retardant, and growth retardant (Pallarés et al., 2017; Sedova et al., 2018). The International Agency for Research on Cancer (IARC) has declared both AFB_1_ and the TAF as group 1 category carcinogens (IARC, 2012). Based on the toxicity of aflatoxins, most of the countries around the world have established their own maximum permissible limits (MPLs) for aflatoxins in various foodstuffs ranging between 0.05 and 100 μg/kg (Ismail et al., 2018).

The warm and wet climate that is suitable for tea cultivation is also favorable for the growth of aflatoxin producing fungi and the production of their metabolites. Drying of tea leaves is usually performed above 80°C that is also sufficient to decrease the fungal load; however, the already produced aflatoxins will remain as heating is ineffective for the degradation of aflatoxins (Viswanath et al., 2012). Furthermore, the recontamination of aflatoxin producing fungi may occur due to improper handling, storage, packaging, and transportation (Sedova et al., 2018). Aflatoxins in tea are rarely regulated in most countries. The Custom Union countries (Armenia, Belarus, Kazakhstan, Kyrgyzstan, and Russia) have established an MPL of AFB_1_ in raw tea as 5 μg/kg (TRCU, 2011). Argentina has established the MPL of AFB_1_ and TAF in herbal tea infusion as 5 and 20 μg/kg, respectively (Zhang et al., 2018a).

Pakistan is the seventh largest country in terms of per capita black tea consumption. During, 2007–2016, a massive increase of 35.8% per capita tea consumption was recorded in Pakistan mainly due to increase in population and urbanization. The current tea consumption in Pakistan is estimated to be around 172,911 tons, which is expected to reach 250,755 tons in 2027 (Hassan, 2018). Previously, aflatoxin contamination of various foodstuffs including cereals (Lutfullah and Hussain, 2012), chillies (Iqbal et al., 2010), milk and milk products (Iqbal and Asi, 2013), infant formulas (Akhtar et al., 2017), and poultry meat and eggs (Iqbal et al., 2014) have been reported from Pakistan indicating a suitable environment for the growth of fungus responsible for the production of aflatoxins.

Despite being the most popular beverage, no information is currently available to consumers on the level of aflatoxins in black tea in Pakistan. Therefore, the current study aimed to quantify the level of aflatoxins in commercially available branded and non-branded black tea samples and to calculate the estimated daily intake (EDI) and potential health risks of aflatoxins through consumption of black tea. Furthermore, the impact of local tea making process on the transfer of aflatoxins from tea leaves to the tea beverage was also evaluated.

## Materials and Methods

### Sample Collection

A total of 120 black tea samples (90 branded and 30 non-branded) were analyzed for the quantification of TAF. The branded samples were collected in their available commercial packaging and the non-branded samples (available in open form) were collected in clean glass bottles and immediately transported to the laboratories of Institute of Food Science and Nutrition, Bahauddin Zakariya University, Multan. The samples were collected from different retail shops (*n* = 23), supermarkets (*n* = 8), and local tea café (*n* = 13) of Multan city of Pakistan during the period of March 2018–July 2018. Non-branded tea samples being cheaper and even illegal (due to unpacked form) therefore are available only in the small scale local tea café and small retail shops, in supermarkets only branded tea samples are available. All the black tea samples were ground with a Waring blender (West Point, France, WF-7901) to obtain homogenous particle size, dried and stored in air tight glass containers at 4°C until analysis.

### Quantification of Aflatoxins in Black Tea

#### Sample Extraction and Immunoaffinity Clean-Up

Test portions of finely grounded black tea (25 g) were extracted with 100 ml methanol/water (60/40 v/v) in an orbital shaker at 200 r/min for 4–5 h, and the extract was filtered through Whatman filter paper No. 42. The filtrate (4 ml) was diluted with 16 ml 50 Mm PBS (pH = 7.4). The diluted extract was passed through immunoaffinity columns (Eurofins) under gravity with a flow rate of 1–3 ml/min. The column was rinsed with 5 ml of 10 Mm PBS/methanol (90/10 v/v). The bounded toxins were released by elution with a total of 2 ml of methanol. The column elute was dried under nitrogen stream.

#### Derivatization

Post-column derivatization of aflatoxins was performed according to the AOAC official method 2005.08 (AOAC, 2005). Briefly, the dried sample extracts/standards were dissolved in 200 μl hexane. TFA (50 μl) was added and the vial was closed and placed in dark for 5 min. The solution containing aflatoxins was mixed with 1.95 ml mixture of double distilled water and acetonitrile (9:1) and vortexed for 1 min. The aqueous layer (lower layer) containing aflatoxins was removed and filtered through a 0.45 μm syringe filter before loading on HPLC (Sykam, Germany).

#### Chromatographic Analysis

HPLC method for aflatoxins quantification was optimized according to the method given by Santos et al. (2010). An isocratic mobile phase of acetonitrile/methanol/water (15/25/65 v/v/v) was found most optimum with a flow rate of 1.0 ml/min. The temperature of column oven was set at 37°C. The excitation and emission wavelengths of fluorescent detector (Sykam, RF-20A) were set at 365 and 440 nm, respectively. The stationary phase used was a reverse phase silica gel C-18 column. The injection volume was 20 μl and the rum time for each sample and standard was 20 min. The retention times for AFG_1_, AFB_1_, AFG_2_, and AFB_2_ were 4.28, 5.09, 6.66, and 8.78 min, respectively.

#### Method Validity

The recovery percentage of the adopted procedure was computed by spiking aflatoxins free tea samples with three different concentrations of aflatoxins, i.e., 6, 12, 24, 48 μg/kg, the ratios of AFB_1_, AFG_1_, AFB_2_, and AFG_2_ were 1:1:0.5:0.5, respectively. The standards were run independently in six replicates and allowed to stand overnight for its adsorption within the samples. The spiked samples were extracted, passed through immunoaffinity column, derivatized, and run on HPLC according to the procedure as described above and the recovery percentages were calculated by using the following equation:

$$Recovery\left(\%\right)=\frac{m\,e\,a\,s\,u\,r\,e\,d\,c\,o\,n\,c\,e\,n\,t\,r\,a\,t\,i\,o\,n\,s}{s\,p\,i\,k\,e\,d\,c\,o\,n\,c\,e\,n\,t\,r\,a\,t\,i\,o\,n}\times 100$$

### Impact of Local Tea Making Process on the Transfer of Aflatoxins

Black tea locally known as “*Chai*” is made in Pakistan by boiling grounded tea leaves in water, then milk and sugar are added and again boiled. The tea is boiled four to five times to give the proper taste of tea leaves. Finally, the tea is filtered through tea strainers and poured in tea cups of normally 150 ml serving per cup. On an average, 5 g tea leaves are added to make one serving of tea (150 ml); however, it may vary person to person based on liking and disliking, ranging between 2.5 and 8 g/serving. Black tea samples detected negative for aflatoxins were used to make tea beverage. Aflatoxins free black tea samples (5 g) were spiked with three different levels of aflatoxins (50, 100, and 200 μg/kg) and allowed to stand for 3 h for complete adsorption of aflatoxins in the tea leaves and for the evaporation of solvent (methanol). The ratio of AFB_1_, AFB_2_, AFG_1_, and AFG_2_ in the spiked samples was 2:1:2:1, respectively (commercially available standard ratio). Tea leaves (5 g) were boiled in 50 ml of water and added with 200 ml of milk and were added with 5 g of sugar. The whole mixture was again boiled four times and then filtered with tea strainer (30 mesh size). The boiling was performed in air tight electric kettle and the kettle was opened after 5–7 min to avoid the removal of water vapors. The tea residues and the tea beverage (filtrate) were dried and analyzed to quantify the concentration of aflatoxins. Non-spiked and spiked unprocessed samples were run as −ive and +ive controls, respectively. Milk and sugar samples were also quantified for the presence of TAF.

### Exposure of Aflatoxins and Health Risk Assessment

A survey was conducted to estimate the daily intake of tea by the male (*n* = 125) and female (*n* = 125) residents of Multan city of Pakistan, aging above 20 years. For exposure assessment of aflatoxins through tea consumption, the “consumer only” scenario for both male and female groups of consumers was considered, i.e., only tea lover male and females were added in the study. A *pro forma* was designed in which individual weight and intake level of tea on daily basis were inquired. The mean values of intake rate and average weight were computed for male and female groups. The EDI of aflatoxins through tea consumption was computed by using the following equation of the Food and Agriculture Organization [FAO]/World Health Organization [WHO] (2014):

$$\mathrm{EDI}=\frac{\left(\mathrm{IR}\times C\right)}{W{}_{\mathrm{AB}}}$$

Where EDI = ng/kg bw/day; IR = intake rate of black tea (kg/person/day), C = the average concentration of aflatoxins detected in tea samples (μg/kg), and W_AB_ = the average body weight of an individual (kg).

To estimate the potential health risk related to the consumption of non-branded and branded black tea, the margin of exposure (MOE) using benchmark dose (BMD) approach of the European Food Safety Authority was used. For dose–response modeling of aflatoxins, the BMD lower confidence limit (BMDL_10_) for a 10% increase in cancer incidence obtained from animal study data modeling (170 ng/kg bw/day) was considered (European Food Safety Authority [EFSA], 2007). The value of MOE for both adult male and female population groups was calculated using the following equation:

$$M\,O\,E=\frac{BMDL{}_{10}}{E\,x\,p\,o\,s\,u\,r\,e}$$

MOE ≥ 10,000 indicates a low public health risk associated with exposure to a genotoxic carcinogen (European Food Safety Authority [EFSA], 2005; Wang et al., 2018).

### Statistical Analysis

Statistical analysis of data was performed by using Statistix 8.1 software (Informer Tech. Inc., United States). The probability level of <0.05 was considered as statistically significant. Mean and standard deviation (±) values were computed by using Microsoft Excel, 2013.

## Results and Discussion

### Occurrence of Aflatoxins in Non-branded and Branded Tea

The efficiency of the HPLC method adopted for the quantification of aflatoxins in black tea samples is presented in Table 1. The recovery percentages for different types of aflatoxins ranged between 85.0 and 96.3%, while the variation coefficient for aflatoxins ranged from 3.9 to 12.5%. The LOD values for AFB_1_ and AFG_1_ were 0.06 and 0.02 μg/kg for AFB_2_ and AFG_2_, while the LOQ value for AFB_1_ and AFG_1_ were 0.18 and 0.06 μg/kg for AFB_2_ and AFG_2_.

**TABLE 1:** HPLC method efficiency and validity (*n* = 6 for each spiking level).

Type of AF	Level of spiking (μg/kg)	Mean values found (μg/kg)	Standard deviation	Recovery (%)	Variation coefficient (%)
AFB_1_	2	1.9	0.1	95.1	5.3
	4	3.7	0.2	92.5	5.4
	8	7.7	0.3	96.3	3.9
	16	15.1	0.8	94.4	5.3
AFG_1_	2	1.8	0.1	90.0	5.6
	4	3.7	0.3	92.5	3.9
	8	7.6	0.3	95.0	4.0
	16	15.2	0.7	95.0	4.6
AFB_2_	1	0.9	0.1	91.1	8.9
	2	1.7	0.2	85.0	11.7
	4	3.6	0.2	90.0	5.6
	8	7.1	0.6	88.8	8.5
AFG_2_	1	0.8	0.1	85.0	12.5
	2	1.7	0.2	88.1	11.7
	4	3.5	0.3	88.5	8.6
	8	7.2	0.5	90.6	6.9

Results for TAF concentrations in non-branded and branded tea samples are represented in Table 2. Aflatoxins were recorded as positive in 94 samples of black tea (78.3%). TAF concentration in the positive samples ranged between 0.11 and 16.17 μg/kg. AFB_1_ was detected as positive in 76.7% (*n* = 92) samples of black tea. The concentration of AFB_1_ in positive samples ranged between 0.08 and 8.24 μg/kg. Statistical analysis (factorial design) revealed significant difference between the concentration of aflatoxins in different types of branded and non-branded tea samples (*P* < 0.05). Aflatoxins were found positive in 93% non-branded samples while Brands A, B, and C were found to have 73, 90, and 57% samples positive for aflatoxins. As there is no set limit for the levels of aflatoxins in tea, the results of the study were compared with the MPL of TAF (10 μg/kg) and AFB_1_ (5 μg/kg) as set by European Union in spices (EU, 2006). Seven black tea samples (five non-branded and two branded) were found to have AFB_1_ level above 5 μg/kg, while six samples of black tea (four non-branded and two branded) were found to have TAF above 10 μg/kg. However, the mean concentration of aflatoxins in all types of branded samples as well as in non-branded samples was below 5 μg/kg, indicating a low level of aflatoxins in black tea samples from Pakistan.

**TABLE 2:** Concentration of total aflatoxins (μg/kg) in branded and non-branded tea samples (*n* = 30 for each type).

Type	AFB_1_ (mean/% + ive)	AFB_2_ (mean/% + ive)	AFG_1_ (mean/% + ive)	AFG_2_ (mean/% + ive)	TAF (mean/% + ive)
Brand A	0.41±0.39^3^/73	0.12±0.25^bc^/40	0.01±0.05^b^/7	0.01±0.04^b^/10	0.55±0.51^c^/73
Brand B	1.67±1.75^b^/90	0.38±0.70^b^/47	0.11±0.33^ab^/20	0.17±0.56^ab^/13	2.32±2.94^b^/90
Brand C	0.48±0.63^c^/53	0.05±0.11^c^/17	0.01±0.03^b^/10	0.01±0.04^b^/10	0.55±0.67^c^/57
Non-branded	2.67±2.20^a^/90	0.91±0.97^a^/80	0.24±0.55^a^/27	0.40±0.92^a^/37	4.22±4.08^a^/93

*Means values having different letters within a column are significantly different from each other (*P* < 0.05); values after ± indicate standard deviation.*

Prevalence of aflatoxins in different types of tea leaves and tea beverages has been reported by a number of researchers from around the world. However, only a few reports are available regarding the levels of aflatoxins in black tea. Pouretedal and Mazaheri (2013) analyzed the concentration of aflatoxins in 40 samples of raw black tea collected from Iranian markets and found 27.5% (*n* = 30) samples positive for aflatoxins, while the mean concentrations of AFB_1_ and TAF were 10 and 12.07 μg/kg, respectively. In China, the mean concentration of AFB_1_ in the samples of raw pu-erh tea and ripe pu-erh tea was 8.33 and 20.149 μg/kg, respectively (Li et al., 2015). Mannani et al. (2019) investigated the concentration of aflatoxins in herbal green tea samples collected from the Moroccan market. Out of 129 analyzed samples, 76 samples were found positive for aflatoxins ranging between 1.8 and 116.2 μg/kg. Pallarés et al. (2017) quantified the levels of aflatoxins in 12 samples of black tea bags from Spain and found that AFB_1_, AFB_2_, and AFG_1_ were absent in all of the analyzed samples while only two samples were contaminated with AFG_2_ with concentration below LOQ (0.5 μg/kg). Comparing our results with the already published literature, it can be stated that the levels of aflatoxins in black tea samples obtained in the current study are lower than those reported from Morocco, in line with the findings from Iran and China but higher than those reported from Spain. Lower levels of aflatoxins in the branded black tea samples as compared to non-branded samples indicate the adoption of good hygienic practices, proper implementation of rules and regulations, and provision of proper storage and transportation facilities. Higher levels of aflatoxins in non-branded spices as compared to branded spices are reported by Naz et al. (2016) and Akhtar et al. (2020). The relatively higher levels of aflatoxins in non-branded tea samples might be due to their sale in unpacked/open form that provides more chances for the fungus to grow and to produce aflatoxins in black tea. The unpacked tea samples are usually stored in bulk until being supplied to markets, the bulk storage facilitates moisture migration and as a result increases the chances of mold growth any mycotoxins production (Chen et al., 2018). The prevalence of aflatoxins in line with this study is already reported in various foodstuff of Pakistan, for instance, in milk samples (Ismail et al., 2016), in animal feed samples (Ismail et al., 2017), and in mother milk samples (Khan et al., 2018) indicating that the environmental conditions in Pakistan are quite favorable for the growth of fungus and the production of aflatoxins.

### Exposure Assessment and Health Risk Characterization of Aflatoxins

The EDI values for AFs due to the consumption of non-branded and branded black tea are given in Table 3. Male group of consumers was found to be more susceptible to the exposure of aflatoxins through consumption of contaminated tea than the female group of consumers due to the higher consumption rate of tea beverage by male group of consumers (0.009 kg/person/day) compared to the female group (0.006 kg/person/day). The consumption rate of non-branded tea (0.008 kg/person/day) was higher than the branded tea (0.007 kg/person/day). The estimated mean exposure to TAF through consumption of non-branded and branded tea beverage were 0.544 and 0.129 ng/kg bw/day, respectively. Owing to the genotoxic and carcinogen nature of aflatoxins, there is no consensus for tolerable daily intake of AFs and therefore the approach of “As Low as Reasonably Achievable” (ALARA) is usually adopted against aflatoxins. However, the provisional maximum tolerable daily intake (PMTDI) value of aflatoxins for children and adults without hepatitis is 1 ng/kg bw/day (Kuiper-Goodman, 1998; Jecfa., 2017). Based on the results obtained from the present study, none of the exposure levels exceeded the reported limit. Sedova et al. (2018) calculated the dietary exposure to AFs through consumption of tea based on combined data from several countries (including Iran, Korea, and Russia) using lower bound approach. The estimated dietary exposure to aflatoxins through tea consumption was reported ranging between 0.4 and 2.6 ng/kg bw/day (mean 0.16 ng/kg bw/day), that is almost in line with our findings. The findings of this study indicate that there is a clear difference in the concentration of aflatoxins in non-branded (< LOD—16.17 μg/kg) and branded tea samples (< LOD—11.07 μg/kg). The non-branded samples were found to have higher aflatoxins level as compared to any of the analyzed brands of tea. Although the EDI values through intake of non-branded tea are less than the PMTDI value but still, the chances of toxicity are there especially for the consumers having large intake of tea on a regular basis.

**TABLE 3:** Estimated dietary exposure (ng/kg bw/day) and MOE values of total AFs for the general adult population.

Consumer Groups	Non-branded tea				Branded tea			
		
	Number of consumers	Average CR^a^	Exposure^b^	MOE	Number of consumers	Average CR^a^	Exposure^b^	MOE
Male	75	0.010	0.680	250	50	0.008	0.147	1156.5
Female	75	0.006	0.408	416.7	50	0.006	0.110	1545.5

*^*a*^Consumption rate.^*b*^Based on mean body weight of the general population of 62 kg.*

The MOE values obtained from mean dietary exposure to aflatoxins were used to estimate the possible health concerns arising from the consumption of black tea. The MOE values for consumers of both non-branded and branded tea obtained in the current study (range 250–1545.5) are much lower than the safe limit of ≥10,000, thus indicating a potential public health risk. Mean maximum MOE value (1545.50) was recorded for the female group of branded tea consumers while mean minimum MOE value (250) was recorded for the male group of non-branded tea consumers, thus indicating a high health risk in all groups of consumers. To the best of our knowledge, not a single study is published on the estimation of MOE values through consumption of aflatoxin contaminated black tea. In a study conducted by Sakin et al. (2018), the EDI of aflatoxins through Surk (a Turkish dairy food) was estimated to be 0.057 and the mean MOE values for AFB_1_ was reported as 2982. Andrade et al. (2013) reported the MOE values for Brazilian population based on 942 food items, the MOE values ranged between 6 and 25. Heshmati et al. (2017) reported MOE values ranging between 1417 and 4250 from Iran based on the intake of aflatoxins through consumption of dried fruits. MOE values obtained in the current study are in line with the other studies reported from Turkey, Brazil, and Iran that are far below the safe limit of 10,000, thus indicating a severe health risk that needs proper consideration by the health and regulatory agencies.

### Effect of Processing on Aflatoxins in Tea

TAFs were spiked in three different levels (50, 100, 200 μg/kg) to evaluate the impact of tea making process on aflatoxins. The concentration of TAFs in tea residues (collected on tea strainer) and in tea filtrate (beverage) are described in Table 4. All four aflatoxins in milk and sugar were found below the detection limits. The mean recovery percentages of aflatoxins in the analyzed three spiking levels ranged between 55 and 60%. The percentages of aflatoxins recorded in the tea residues obtained on the surface of tea strainer ranged between 3 and 6% while TAF% in the filtrate ranged between 50 and 55%. The results clearly indicate that more than half of the aflatoxins found in tea leaves get dissolved in the water and become a part of the beverage, to be consumed by the tea drinkers. A small percentage of aflatoxins were retained by the tea leaves while around 40–45% of the aflatoxins could not be detected. It is not clear why this is but the results show a large proportion of aflatoxins reach the final beverage.

**TABLE 4:** Aflatoxins recovery in tea residues and filtrate (beverage).

Level of spiking (μg/kg)	TAF in tea residues (μg/kg)	TAF in filtrate (μg/kg)	Mean recovery (%) through tea residues and filtrate
50	1.4 ± 0.2	26.9 ± 2.2	56.6
100	4.6 ± 0.3	55.8 ± 3.2	60.4
200	12.4 ± 1.8	98.9 ± 4.3	55.7

*TAF = total aflatoxins.*

Aflatoxins are water soluble (10–20 mg/mL) and so get easily dissolved in water when the tea leaves are brewed (Sedova et al., 2018). However, a lower percentage of aflatoxins in tea leaves (about 30%) were transferred to the water in a study by Viswanath et al. (2012). The possible reasons behind the differences in results might be due to the difference of tea varieties used in our study as compared to India, or due to the difference in tea making processes, as in this study we added milk and sugar also (local tea recipe), while in the study of Viswanath et al. (2012), the tea leaves were boiled in water only. Low recovery rates of TAFs in case of tea processing might be due to the conversion of aflatoxins in some other metabolites/degradation products due to four to five times boiling of tea. Reduction in aflatoxins level due to heating is reported by a number of authors. Lee et al. (2015) reported 42–81% reduction in aflatoxins level by heating the soybeans at temperature ranging between 100 and 150°C. Rastegar et al. (2017) reported 93% reduction in aflatoxins level (spiking level was 268 μg/kg) of roasted pistachio nuts. However, a number of other researchers have reported the ineffectiveness of heating on aflatoxin (Awasthi et al., 2012; Bohloli-Oskoii and Mohamadi, 2013; Hassan and Kassaify, 2014). Therefore, further studies are required to investigate the presence of possible aflatoxin breakdown products in black tea.

## Conclusion

The current study is the first of its kind that provides information regarding the levels of aflatoxins in commercially branded and non-branded black tea samples available in the local markets of Pakistan. Among the analyzed samples of tea, non-branded black tea samples were found to be more contaminated with aflatoxins as compared to branded samples indicating the adoption of poor post-harvest practices in case of non-branded black tea. Based on the limited number of samples especially of non-branded tea samples, it is not possible to state that the non-branded tea samples are always more contaminated than the branded ones but it is clear that the contamination of aflatoxins in both types of tea samples is common in Pakistan. The MOE values for aflatoxins through black tea consumption indicate severe health risks through black tea consumption. Male consumers are more at risk as compared to female consumers. The study on the impact of the local tea making process on aflatoxins content indicates the transfer of more than 50% aflatoxins from tea leaves to the tea beverage and possible production of aflatoxins degradation products probably due to four to five times boiling of tea in the local tea making method, while only 3–6% aflatoxin are left in the remaining tea residue on the tea strainer. Current findings indicate that the health and regulatory agencies must put strenuous efforts to reduce the load of aflatoxins in commercially available black tea. However, further studies are needed to clearly understand the mechanism involved in the possible degradation of aflatoxins in local tea making processes.

## Data Availability Statement

All datasets generated for this study are included in the article/supplementary material.

## Author Contributions

All the authors actively participated in research work and write up of the article. Planning of research was done by AI, YG, SA, and MR. Experimentation was performed by AI and IN. The write up performed mainly by AI, YG, and MR while some assistance was also provided by SA, MR, and IN.

## Conflict of Interest

The authors declare that the research was conducted in the absence of any commercial or financial relationships that could be construed as a potential conflict of interest.
